# Effect of pericardial incision on left ventricular morphology and systolic function in patients during coronary artery bypass grafting

**DOI:** 10.1186/s12947-020-00206-1

**Published:** 2020-07-21

**Authors:** Lan-Ting Zhao, Lu Liu, Ping-Ping Meng, Yong-Huai Wang, Meng Li, Jun Yang, Tian-Xiang Gu, Chun-Yan Ma

**Affiliations:** 1 Department of Cardiovascular Ultrasound, The First Hospital of China Medical University, No. 155 Nanjingbei Street, Heping District, Shenyang, 11001 Liaoning China; 2 Department of Cardiac Surgery, The First Hospital of China Medical University, No. 155 Nanjingbei Street, Heping District, Shenyang, 11001 Liaoning China

**Keywords:** Coronary artery bypass grafting, Pericardial incision, Morphology, Global longitudinal strain

## Abstract

**Background:**

Accurate assessment of left ventricular (LV) systolic function is important after coronary artery bypass grafting (CABG). LV ejection fraction (LVEF) is conventionally used to evaluate LV systolic function; deformation parameters can be used to detect subtle LV systolic dysfunction. It is unclear whether an incised pericardium without sutures during CABG could affect LV morphology and function. We investigated the effect of pericardial incision on LV morphology and systolic function during CABG.

**Methods:**

Intraoperative transesophageal echocardiography was performed in 27 patients during elective off-pump beating heart CABG 5 min before and after pericardial incision. LV longitudinal and mid-cavity transversal diameters, sphericity index, volumes, and LVEF were measured. LV global longitudinal strain (GLS), global circumferential strain (GCS), global radial strain (GRS), and twist obtained by two-dimensional speckle tracking echocardiography were measured simultaneously.

**Results:**

LV mid-cavity transversal diameter increased, while the LV sphericity index decreased (*P* < 0.001) immediately after pericardial incision. The GLS, GCS, and twist significantly decreased, while the GRS notably increased (*P* < 0.001). The LV volumes and LVEF remained unchanged.

**Conclusions:**

Pericardial incision immediately transformed LV morphology from an ellipsoid to sphere, with decreased longitudinal and circumferential strain and twist, and increased radial strain, while LVEF remained unchanged. This should be considered when evaluating LV systolic function in patients after CABG.

## Background

As a coronary artery revascularization therapeutic strategy, coronary artery bypass grafting (CABG) can effectively improve the myocardial blood supply for complex coronary artery disease. Echocardiography is an optimal, noninvasive method for precisely evaluating left ventricular (LV) systolic function, which has an important role in estimating the therapeutic effect in patients after CABG.

LV ejection fraction (LVEF) is conventionally used as a marker of LV systolic function. Deformation parameters obtained by two-dimensional speckle tracking echocardiography (2D STE) can sensitively detect subtle LV dysfunction at an early stage, LV global longitudinal strain (GLS), in particular, appears to be a more robust parameter and has incremental value in patients referred for cardiac surgery including CABG [1–6].

The pericardium is often incised during CABG without sutures after the surgery, which may affect ventricular morphology and systolic function. Unsworth et al. [7] reported that pericardial incision induced a reduction in right ventricular (RV) systolic function in 9 patients who underwent CABG; however, it is still unclear whether it can affect LV systolic function. Therefore, we sought to investigate the effect of pericardial incision on LV morphology and systolic function during CABG using intraoperative transesophageal echocardiography (TEE).

## Methods

### Study population

Patients who underwent elective off-pump beating heart CABG between January 2019 and June 2019 in our hospital were consecutive recruited for this study. Patients were excluded if they had a prior history of cardiac surgery, a combination of other cardiac surgery, pericardial disease, arrhythmia, or contraindication to TEE. All patients underwent coronary angiography before CABG and their results were assessed by experienced cardiologist. The SYNTAX score was calculated using the SYNTAX score algorithm (www.syntaxscore.com) [8]. All procedures performed in the studies involving human participants were in accordance with the ethical standards of the Ethics Committee of the China Medical University. Informed consent was obtained from all individual participants included in the study.

### Intraoperative echocardiographic image acquisition

After induction of anesthesia, an intraoperative TEE examination was performed 5 min before and after pericardial incision during the surgery using the CX50 ultrasound system (Philips Healthcare, Andover, MA, USA) equipped with an X7-2t transducer. The TEE probe was inserted into the patient’s esophagus to obtain mid-esophageal 4-chamber (ME 4C), 2-chamber (ME 2C), and long-axis (ME LAX) views, and into the patient’s stomach to obtain transgastric basal, mid-papillary, and apical short-axis views. The basal level was identified by the mitral valve, and the apical level was defined as proximal to the level with ventricular cavity obliteration at end-systole. All manipulations of image acquisition were performed according to the recommendations from the American Society of Echocardiography [9]. Standard two-dimensional cine loops, including at least 3 consecutive cardiac cycles with frequency of 50–70 frames per second, and Doppler spectrum were stored in cine-loop format.

### Echocardiographic image measurements and analyses

All data were transferred to a workstation for further offline analysis using the Qlab software package (Philips Healthcare). LV longitudinal and mid-cavity transversal diameters were measured at end-diastole in ME 4C view, and the LV sphericity index (LVSI) was defined as the ratio of the LV longitudinal to mid-cavity transversal diameter [10]. The LV internal diameter and posterior wall thickness were measured at end-diastole in transgastric mid-papillary short-axis view, and the relative wall thickness was calculated simultaneously [9]. The LV end-diastolic volume, end-systolic volume, stroke volume, and LVEF were measured using the biplane modified Simpson’s method with ME 4C and ME 2C views. Mitral inflow early (mitral E) and late (mitral A) diastolic velocity, mitral annular septal and lateral early diastolic velocity (septal e’ and lateral e’), and tricuspid regurgitation velocity were measured in the ME 4C view. Then mitral E/A, and mitral average E/e’ were calculated.

Deformation parameters, including LV GLS, global circumferential (GCS), and global radial strain (GRS), were measured using 2D STE. Endocardial contour was manually adjusted to optimize tracking when necessary, and patients with inadequate tracking, which was defined by the mismatch between the region of interest and the myocardium during dynamic loops, of more than two segments were excluded from the study. The degree of LV peak systolic apical and basal rotation was measured at the apical and basal levels, respectively. Twist was defined as the net difference between apical and basal rotation. The absolute change rates of LV GLS, GCS, GRS, twist, and LVSI were calculated as the percentage of the difference in these parameters before and after pericardial incision divided by that before pericardial incision. A significant reduction of GLS was defined as a decrease of GLS by > 15% immediately after pericardial incision [11].

The RV basal, mid-cavity transversal, and longitudinal diameters were measured at end-diastole in ME 4C view, and the RV sphericity index was defined as the ratio of the RV longitudinal to mid-cavity transversal diameter [10]. The RV end-diastolic and end-systolic areas were measured in ME 4C view, and the RV fractional area change was calculated simultaneously [1].

### Hemodynamic measurements

Hemodynamic monitors were supplemented by radial arterial and central venous catheters. Hemodynamic parameters including systolic blood pressure (SBP), diastolic blood pressure (DBP), mean arterial blood pressure (MAP), central venous pressure (CVP), and heart rate (HR) were recorded simultaneously at the time of image acquisition.

### Reproducibility

Intra- and inter-observer agreement for global strain and rotation were examined in 10 randomly selected patients. The same observer, who was blinded to the initial measurements, repeated the measurements to assess intra-observer agreement after more than 4 weeks had elapsed. And another independent observer repeated the measurements twice to assess inter-observer agreement.

### Statistical analysis

Statistical analyses were performed using SPSS 25.0 (IBM Corp., Armonk, NY, USA). Normality plots with tests were performed using the Shapiro–Wilk test. Categorical data are presented as frequencies and percentages, normally distributed data as mean ± SD, and non-normally distributed data as median and interquartile range. Comparisons of continuous variables before and after pericardial incision were made using the paired Student t-test or Wilcoxon test, as appropriate. Comparisons of SYNTAX score between patients with and without reduction of GLS by > 15% were made using the independent Student *t*-test. The Pearson coefficient was used for correlation analysis. The relationship between the change rate of GLS and continuous variables was analyzed using simple linear regression analysis. Intra-observer and inter-observer agreement analyses of global strain and rotation were assessed using Bland-Altman analysis in 10 randomly selected patients. A *P*-value < 0.05 was considered significant.

## Results

### Baseline characteristics

**Fig. 1:**
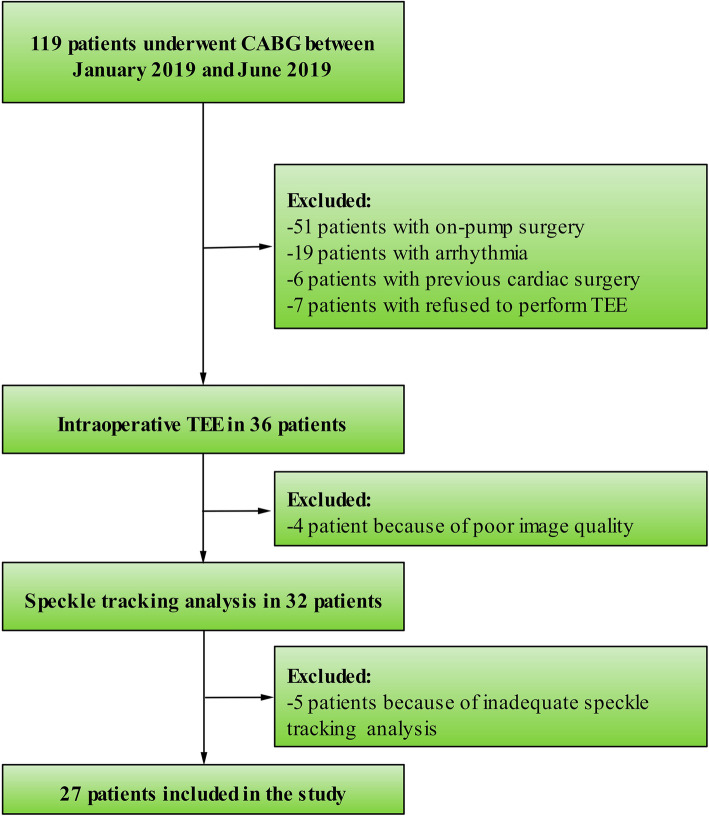
Patient flow chart

A total of 36 patients performed the intraoperative TEE. Among these patients, 4 patients were excluded because the standard transgastric short-axis view with optimal image quality were unable to be acquired, and 5 patients were excluded due to the inadequate speckle-tracking analyses from apical views (*n* = 1) and transgastric short-axis views (*n* = 4). Finally, 27 patients (18 men; mean age, 61.2 ± 6.7 years) were enrolled in the analyses (Fig. 1). During the perioperative period, there were 2 patients experienced prolonged mechanical ventilation due to poor cardiopulmonary function. The baseline characteristics of these patients are shown in Table 1.

**Table 1:** Patient baseline characteristics

Variable	Value (*n* = 27)
Age (y)	61.2 ± 6.7
Male sex	18 (66.7%)
Body mass index (kg/m^2^)	25.76 ± 3.29
Smoker	10 (37.0%)
Medical History	
Hypertension	17 (63.0%)
Diabetes mellitus	8 (29.6%)
Previous myocardial infarction	13 (48.1%)
Dyslipidemia	8 (29.6%)
Pulmonary hypertension	1 (3.7%)
Previous PCI	4 (14.8%)
Peripheral vascular disease	4 (14.8%)
Troponin I level (ng/ml)	0.01 (0.004, 0.03)
Brain-type natriuretic peptide level (pg/ml)	49.00 (21.00, 83.00)
NYHA class (I/II/III/IV)	4 (14.8%)/17 (63%)/6 (22.2%)/0 (0%)
SYNTAX score	29.02 ± 9.53
Euroscore II	1.34 ± 0.64
Medications	
Aspirin	18 (66.7%)
Beta-blockers	23 (85.2%)
Calcium channel antagonists	4 (14.8%)
Statins	21 (77.8%)
Nitrates	24 (88.9%)

Data are expressed as a mean ± standard deviation, median (interquartile range) or frequencies (percentages)

*Abbreviations*: *PCI* percutaneous coronary intervention, *NYHA* New York Heart Association

### Hemodynamics measurements before versus after pericardial incision

Immediately after pericardial incision, there was no significant change in CVP, SBP, DBP, MAP, and HR (Table 2).

**Table 2:** Hemodynamic parameters before and after pericardial incision

Parameter	Before pericardial incision (*n* = 27)	After pericardial incision (*n* = 27)	*P*-value
Heart rate (beats/min)	60.67 ± 14.05	64.56 ± 10.90	0.15
Central venous pressure (mmHg)	11.07 ± 1.88	10.78 ± 1.28	0.06
Systolic blood pressure (mmHg)	124.67 ± 12.89	122.93 ± 8.83	0.18
Diastolic blood pressure (mmHg)	69.37 ± 6.22	68.56 ± 5.44	0.16
Mean arterial pressure (mmHg)	87.80 ± 6.29	86.68 ± 4.19	0.15

Data are expressed as a mean ± standard deviation

### LV morphology and function before versus after pericardial incision

The LV mid-cavity transversal diameter increased (*P* < 0.001), while the LV sphericity index significantly decreased immediately after pericardial incision (*P* < 0.001), and the posterior and relative wall thicknesses increased (*P* < 0.05, Table 3). There were no significant differences in mitral E, A, E/A, septal e’, lateral e’ and mitral average E/e’, and tricuspid regurgitation velocity between before and after pericardial incision (Table 3).

**Table 3:** Changes in LV morphology and function before and after pericardial incision

Parameter	Before pericardial incision (*n* = 27)	After pericardial incision (*n* = 27)	*P*-value
LV longitudinal diameter (mm)	71.48 ± 7.48	70.19 ± 8.00	0.20
LV mid-cavity transversal diameter (mm)	36.74 ± 3.99	41.41 ± 4.59	**<0.001**
LV sphericity index	1.96 ± 0.26	1.70 ± 0.19	**<0.001**
LV internal diameter (mm)	44.65 ± 6.06	45.10 ± 6.42	0.64
Posterior wall thickness (mm)	7.59 ± 1.06	8.13 ± 0.82	**0.007**
Relative wall thickness (cm)	0.34 ± 0.06	0.37 ± 0.06	**0.04**
Mitral E (m/s)	0.66 ± 0.20	0.61 ± 0.18	0.17
Mitral A (m/s)	0.87 ± 0.18	0.83 ± 0.17	0.18
Mitral E/A	0.81 ± 0.38	0.77 ± 0.29	0.47
Septal e’ (cm/s)	6.26 ± 2.15	6.04 ± 2.10	0.16
Lateral e’ (cm/s)	7.66 ± 2.81	7.47 ± 2.88	0.10
Mitral average E/e’	10.38 ± 3.62	10.16 ± 3.79	0.58
Tricuspid regurgitation velocity (m/s)	1.52 ± 0.61	1.47 ± 0.59	0.43
LV end-diastolic volume (mL)	109.48 ± 35.05	110.33 ± 35.33	0.25
LV end-systolic volume (mL)	47.85 ± 22.12	49.04 ± 22.58	0.26
LV stroke volume (mL)	61.63 ± 15.06	61.30 ± 15.85	0.74
LVEF (%)	57.41 ± 6.08	56.63 ± 6.82	0.35
GLS (%)	−15.86 ± 2.66	−12.39 ± 3.27	**<0.001**
GCS (%)	−22.25 ± 4.56	−18.45 ± 5.07	**<0.001**
GRS (%)	18.41 ± 6.05	23.13 ± 7.32	**<0.001**
Apical rotation (°)	3.67 ± 1.69	1.63 ± 0.70	**<0.001**
Basal rotation (°)	−4.14 ± 1.04	−3.08 ± 1.12	**<0.001**
Twist (°)	7.81 ± 2.12	4.71 ± 1.22	**<0.001**

Data are expressed as mean ± standard deviation

*Abbreviations*: *LV* left ventricular, *E* mitral inflow early diastolic velocity, *A* mitral inflow late diastolic velocity, *e’* mitral annular early diastolic velocity, *LVEF* left ventricular ejection fraction, *GLS* global longitudinal strain, *GCS* global circumferential strain, *GRS* global radial strain

**Fig. 2:**
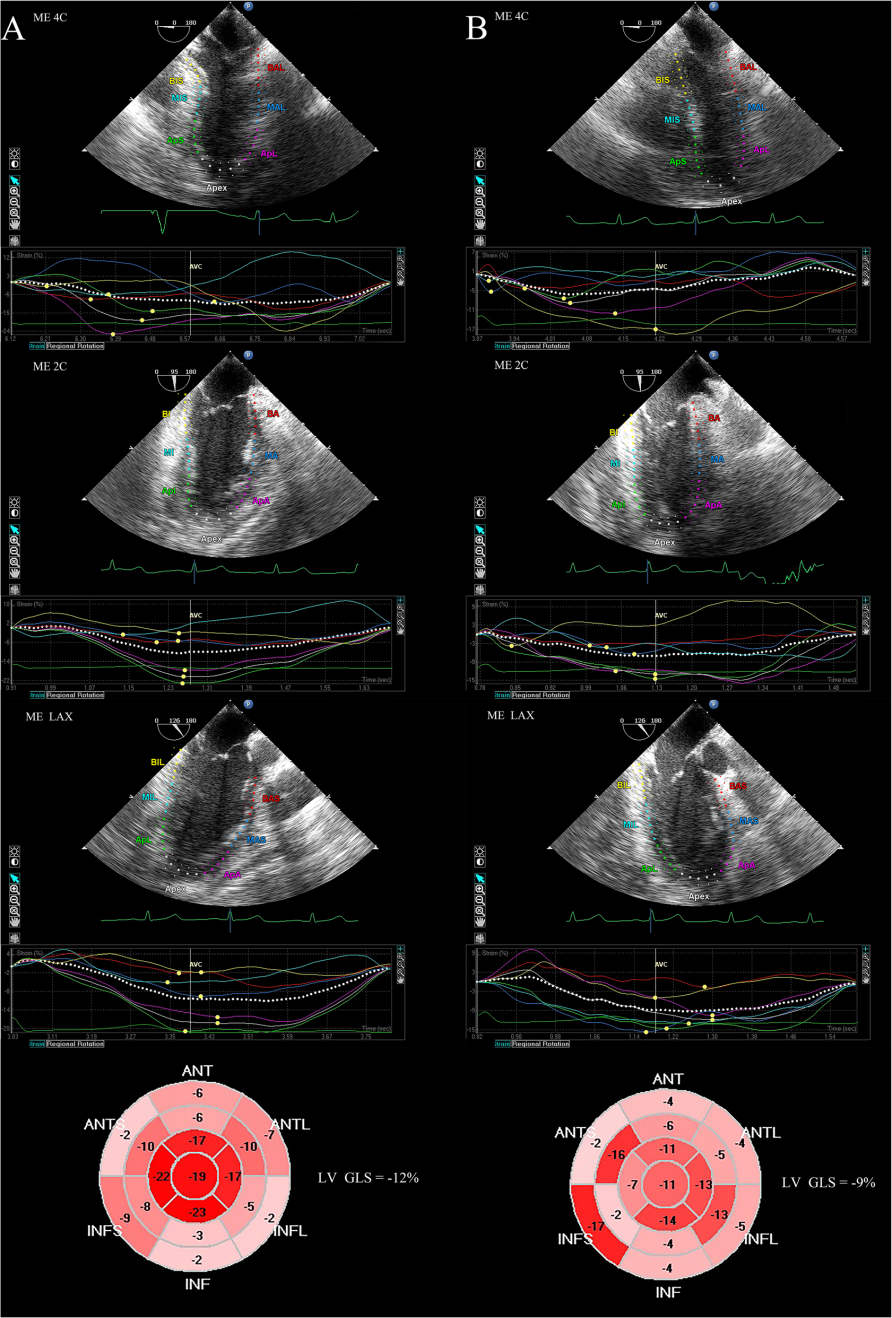
Measurement of longitudinal strain in ME 4C, ME 2C, and ME LAX view before (**a**) and after (**b**) pericardial incision in a case with ischemic cardiomyopathy. This patient had three vessel coronary artery lesions with chronic total occlusion in left circumflex artery. ME 4C, mid-esophageal 4-chamber; ME 2C, mid-esophageal 2-chamber; ME LAX, mid-esophageal long-axis; LV, left ventricular; GLS, global longitudinal strain

In the 27 patients, there were 16 (9.9%) views were manually adjusted to optimize the tracking of LV myocardium. After pericardial incision, LV GLS and GCS decreased, and the GRS increased simultaneously (*P* < 0.001), while the LV volumes and LVEF remained unchanged. Meanwhile, the reduction of LV twist was accompanied by decreased apical and basal rotation (*P* < 0.001, Table 3 and Fig. 2). Furthermore, the change rate of GRS was correlated with the change rate of GCS (*r* = 0.44, *P* = 0.02), and the change rate of GLS was significantly and inversely correlation with the SYNTAX score (*r* = − 0.63, *P* < 0.001).

Seventeen patients had a significant reduction of GLS by > 15% immediately after pericardial opening, and the SYNTAX score was lower in these patients than in the other 10 patients with nonsignificant variability of GLS (24.09 ± 6.03 versus 37.40 ± 8.59, *P* < 0.001).

### RV morphology and systolic function before versus after pericardial incision

The RV mid-cavity transversal diameter significantly increased and the RV sphericity index decreased (*P* < 0.05), while the RV fractional area change remained unaltered, which indicated that the right ventricle became more round in shape (Table 4).

**Table 4:** Changes in RV morphology and systolic function before and after pericardial incision

Parameter	Before pericardial incision (*n* = 27)	After pericardial incision (*n* = 27)	*P*-value
RV basal diameter (mm)	33.89 ± 3.15	35.37 ± 3.97	0.11
RV mid-cavity transversal diameter (mm)	28.59 ± 2.89	30.44 ± 3.25	**<0.001**
RV longitudinal diameter (mm)	59.89 ± 7.09	59.22 ± 8.59	0.54
RV sphericity index (mm)	2.11 ± 0.29	1.96 ± 0.31	**0.03**
RV end-diastolic area (cm^2^)	13.83 ± 1.56	13.88 ± 2.51	0.88
RV end-systolic area (cm^2^)	7.76 ± 1.09	7.88 ± 1.64	0.68
RV fractional area change (%)	43.78 ± 5.39	43.00 ± 7.47	0.60

Data are expressed as a mean ± standard deviation

*Abbreviations*: *RV* right ventricular

Results of the intra-observer and inter-observer agreement analyses of global strain and rotation are presented in Table 5.

**Table 5:** Intra-observer and inter-observer agreement analyses of global strain and rotation

Parameter	Intra-observer agreement analysis		Inter-observer agreement analysis	
	Bias	95% LOA	Bias	95% LOA
GLS	−0.17	−2.94-2.60	−0.21	−3.08-2.65
GCS	0.22	− 3.02-3.47	−0.36	−6.02-5.29
GRS	0.19	−3.72-4.10	0.24	−3.74-4.23
BR	0.33	−2.56-3.21	0.44	−2.52-3.41
AR	0.31	−2.63-3.26	0.55	−2.93-4.04

*Abbreviations*: *LOA* limits of agreement, *GLS* global longitudinal strain, *GCS* global circumferential strain, *GRS* global radial strain, *GRS* global radial strain, *BR* basal rotation, *AR* apical rotation

## Discussion

In the present study, we found that the pericardial incision can immediately induce the obvious change of LV morphology and contractile pattern. LV morphology changed from an ellipsoid to a sphere, and the relative wall thickness increased immediately after pericardial incision. Additionally, LV deformation parameters markedly changed with a reduced LV global longitudinal and circumferential strain and twist, and an increased global radial strain; however, LV volumes and ejection fraction remained unchanged.

The pericardium maintains the anatomic position of the heart and limits its displacement through ligaments; besides, the relatively inelastic parietal pericardium retracts when it is cut, indicating that it exerts pressure on the underlying myocardial chambers [12–14]. Therefore, pericardial incision may alter the geometry or function of heart during cardiac surgery. Currently, in addition to the patients with CABG, the pericardium is also not sutured after the surgery in the patients with pericardiectomy and inadequate pericardial tissue for suturing, such as adopting autologous pericardial patches in aortic dissection, possible cardiac enlargement due to myocardial edema in complex congenital heart disease or severe valvular heart disease, and so on. Several previous studies have reported a change of RV geometry or reduction of RV systolic function after pericardial incision without suture [7, 15–17], which was similar with our study. Interestingly, the study by Zanobini M, et al. [18] also reported the presence of decreased RV systolic function after pericardial incision even with reclosing the pericardium with a continuous suture in different surgical approach and cardioplegia type. The possible mechanisms of decreased RV function after cardiac surgery may be related to the cardiopulmonary bypass use, pericardial adhesions, modality of cardioplegia delivery and pericardial incision, and so on [18], and its clear-cut mechanisms were warranted to be further addressed in the future study.

To date, the existing researches has mainly focused on the influences of pericardial incision on RV. However, only a few studies have researched the effect of the pericardium on LV morphology and systolic function. Tanaka et al. [19] reported that congenital pericardial defects caused depressed apical and basal rotation, and consequently, LV twist was reduced. A canine experiment also showed that twist was reduced after pericardial opening and restored after suturing the pericardium, which suggests the importance of the pericardium in sustaining LV twist [20]. It is still unclear whether pericardial incision could affect LV morphology and systolic function during CABG.

Our study revealed the change of LV morphology from an ellipsoid to sphere after pericardial incision in the patients with CABG, as well as the reduced LV global longitudinal and circumferential strain, reduced twist and increased global radial strain. In the normal heart, pericardial incision may lead to the change of LV preload, filling pressure, and blood pressure [21]. However, these responses may not be so evident in the heart with severe myocardial damage caused by the stenosis or occlusion of coronary artery. Although our results showed the subtle changes between before and after pericardial incision in CVP, SBP, DBP, MAP, and HR with invasive methods, as well as LV filling pressure by noninvasive echocardiography in the patients with CABG, there were no significant differences for all of them. Therefore, we infered that the mechanisms underlying the change of LV morphology from an ellipsoid to a sphere and LV contractile pattern might be mainly attributed to the physical release of pericardial constraint. Maybe, the change of pressure was another pathophysiological mechanism; however, these speculations should be further verified in the future study.

The LV myocardium is made up by the three-dimensional arrangement of the myocardial cells, which are supported loosely within a continuous matrix of fibrous tissue. Groups of myocytes are surrounded by condensations of the endomysial weave, thus forming the perimysium, which aggregates a meshwork of myocytes into the so-called myofibers [22, 23]. For describing the global arrangement, most studies have depicted LV myocardium as a transmural continuum which changes continuously from a right-handed helical subendocardium to a left-handed helical subepicardium with the mid-myocardium has a circumferential orientation [24, 25]. The unique structure of myofibers results in complicated myocardial motion during systole, and speckle tracking-derived strain could be used to evaluate ventricular systolic function by detecting myocardial deformation in longitudinal, circumferential, radial direction and wringing from base to apex [26].

Deformation parameters change in different directions during the progression of myocardial disease, however, its instantaneous change in this investigation could not be attributed to this. The paradoxical phenomenon in our study that decreased longitudinal and circumferential shortening with preserved ejection fraction is commonly seen in patients with hypertension [27–29]. As the afterload increased, longitudinal and circumferential shortening decreased while the wall thickened as a compensation allowing a preserved ejection fraction. However, there was no significant hemodynamic change immediately after pericardial incision in our study, hence we speculated that the change in myocardial contractile pattern may mainly result from the physical release of pericardial constraint.

Left ventricular ejection fraction is determined by both myocardial strain and wall thickness, which can increase with the increased strain and wall thickness, and even if the strain decreases, it could keep constant because of increased wall thickness [30–32]. Wall thickness is determined by end-diastolic wall thickness and radial thickening (ie radial strain), it has a substantial influence on ejection fraction: a 1 cm increase in thickness increased ejection fraction by approximately 13 percentage points [31, 32]. Sheet dynamic and sheet geometry between adjacent myocytes and matrix coupling are crucial for LV wall thickening and dynamics, regional ventricular wall thickening also reflects changes in cardiac fiber and sheet structure during contraction [33, 34]. The present study revealed the immediate changes of LV morphology and deformation but preserved LVEF after pericardial incision, which may indicate that LVEF may be more reliable than GLS to immediately evaluate LV systolic function after pericardial incision for cardiac anesthesiologists.

We could not provide a definite mechanism for the change in contraction pattern in our study, we speculated that the variations in sheet dynamic and geometry caused by the changes in the arrangement of myocytes due to lack of pericardial constraints may play a potential role. Although we did not find significant correlation between wall thickness and ejection fraction, we found the decreased circumferential strain was corelated with the increased radial strain. Therefore, we speculated that the increased radial strain may offset the reduction in longitudinal and circumferential shortening and twisting, allowing for an unchanged ejection fraction.

LV global longitudinal strain as a valuable parameter of LV systolic function has been used in clinical application with a better reproducibility, and it could be a predictor of prognosis and therapeutic effect in patients after CABG [1–4, 35, 36]. In this study, a significant reduction of global longitudinal strain was found in 17 patients immediately after pericardial incision. The SYNTAX score of these 17 patients was significantly lower than that of the other 10 patients, probably because patients with a lower SYNTAX score may have few coronary lesions and more surviving myocardia with better movement; therefore, they may be more susceptible to pericardial incision than patients with a higher SYNTAX score.

Transesophageal 2D STE was used to evaluate LV systolic function during CABG in our study, which showed a high degree of intra-observer and inter-observer agreement. Intraoperative TEE is one of the cornerstones for evaluating immediate changes in myocardial function and helps provide critical information during cardiac surgery. Myocardial strain imaging derived from speckle tracking using TEE is a feasible, reproducible method for assessing LV deformation, which has good agreement with transthoracic strain imaging [37–40].

In the present study, LV morphology and systolic function were investigated only immediately after pericardial incision, and a long-term follow-up was not performed to assess whether any aspect of recovery of contractile patterns would occur because we could not eliminate the interference of myocardial revascularization after CABG. However, the depressed RV systolic function after pericardial incision did not recover within 1 month after CABG in patients without interference of the right coronary artery, [7] suggesting that the effect of pericardial incision on RV systolic function may have persisted. Gozdzik A et al. in their study, observed that global longitudinal strain was still reduced 2 years after CABG [41]. Thus, we speculate that the altered LV morphology and contractile pattern may also persist after CABG, and a well-designed animal experiment should be performed in the future.

### Study limitations

This study has several limitations. First, the correlation between the lower SYNTAX score and the reduction of GLS > 15% was analyzed based on the limited sample size in the present study. Therefore, a further study with adequate samples from multicenter should be designed to verify these findings. In addition, the invasive LV filling pressure and pulmonary capillary wedge pressure were unable to be obtained during the off-pump beating heart CABG, which may provide the pathophysiological basis for LV modification. However, we provided the LV diastolic indices by noninvasive echocardiography, such as mitral E, mitral A, E/A, septal e’, lateral e’ and average E/e’, and tricuspid regurgitation velocity, which can indirectly reflect the LV filling pressure. Furthermore, we measured the tricuspid regurgitation velocity using ME 4C, and its value may be influenced by the angle of ultrasound beams and may be different from that measured by TTE; however, the present study highlighted its change after pericardial incision and the difference may not influence the research objective. Moreover, it is also worth mentioning that we obtained the results of this work with the Qlab software, which was originally developed for transthoracic images. The absolute values of strain and rotation in the present study may not be identical to the values by transthoracic echocardiography. Currently, there are no recognized standards for the definition of a reduction in LV global systolic function using GLS. We referred to the standard on cancer therapy from the American Society of Echocardiography, which recommended that a reduction in GLS > 15% from baseline is very likely to be abnormal during chemotherapy. The results should be further analyzed using special standards in the future.

## Conclusions

Pericardial incision immediately transformed LV morphology from a prolate spheroid to a sphere during CABG, the LV contractile pattern changed with a decrease in longitudinal and circumferential shortening and twisting, and radial thickening increased despite an unchanged ejection fraction. The altered global longitudinal strain resulting from pericardial incision should be taken into account when evaluating LV systolic function in patients after CABG, especially in patients with a lower SYNTAX score.

## Data Availability

The datasets used and/or analyzed during the current study are available from the corresponding author on reasonable request.

## Abbreviations

LV: Left ventricular
RV: Right ventricular
TEE: Transesophageal echocardiography
2D STE: Two-dimensional speckle tracking echocardiography
CABG: Coronary artery bypass graft
LVEF: Left ventricular ejection fraction
GLS: Global longitudinal strain
GCS: Global circumferential strain
GRS: Global radial strain
ME 4C: Mid esophageal 4-chamber view
ME 2C: Mid esophageal 2-chamber view
ME LAX: Mid esophageal long-axis view
TGB: Transgastric basal short-axis view
TGM: Transgastric mid-papillary short-axis view
TGA: Transgastric apical short-axis view
